# Erratum: Phase transitions via selective elemental vacancy engineering in complex oxide thin films

**DOI:** 10.1038/srep24695

**Published:** 2016-04-22

**Authors:** Sang A. Lee, Hoidong Jeong, Sungmin Woo, Jae-Yeol Hwang, Si-Young Choi, Sung-Dae Kim, Minseok Choi, Seulki Roh, Hosung Yu, Jungseek Hwang, Sung Wng Kim, Woo Seok Choi

*Scientific Reports* 6: Article number: 2364910.1038/srep23649; published online: 04012016; updated: 04222016

In this Article, Hosung Yu is incorrectly listed as being affiliated with ‘Department of Physics, Inha University, Incheon 22212, Korea’. In addition, Sung Wng Kim is incorrectly listed as being affiliated with ‘Center for Integrated Nanostructure Physics, Institute for Basic Science (IBS) Sungkyunkwan University, Suwon 16419, Korea’ and ‘Department of Physics, Inha University, Incheon 22212, Korea’.

The correct affiliations are listed below:

Hosung Yu and Sung Wng Kim

Department of Energy Sciences, Sungkyunkwan University, Suwon 16419, Korea.

