# Supplementary material for: Phase transitions via selective elemental vacancy engineering in complex oxide thin films
Source: Sci Rep. 2016 Apr 1;6:23649. doi: 10.1038/srep23649 (PMC4817049; doi:10.1038/srep23649)
Supplement: Supplementary Information [file srep23649-s1.pdf]

Supplementary Information

Phase transitions via selective elemental vacancy engineering in complex oxide thin films

Sang A Lee[1,2], Hoidong Jeong[1], Sungmin Woo[1], Jae-Yeol Hwang[3], Si-Young Choi[4], Sung-Dae Kim[4], Minseok Choi[4], Seulki Roh[1], Hosung Yu[5], Jungseek Hwang[1], Sung Wng Kim[3,5], and Woo Seok Choi[1*]

[1]Department of Physics, Sungkyunkwan University, Suwon, 16419, Korea

[2]Insitute of Basic Science, Sungkyunkwan University, Suwon, 16419, Korea

[3]Center for Integrated Nanostructure Physics, Institute for Basic Science (IBS) Sungkyunkwan University, Suwon 16419, Korea

[4]Materials Modeling and Characterization Department, Korea Institute of Materials Science, Changwon 51508, Korea

[5]Department of Energy Sciences, Sungkyunkwan University, Suwon 16419, Korea

[*]e-mail: choiws@skku.edu.

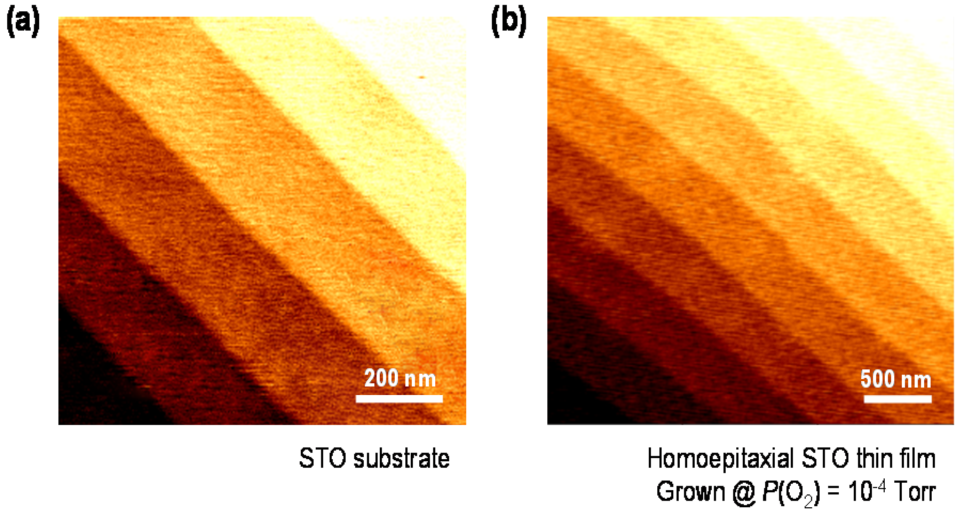

Supplementary Figure S1. Atomic Force Microscopy (AFM) topographic images of (a) $SrTiO_3$ substrate and (b) homoepitaxial $SrTiO_3$ thin film (~100 nm) grown at $P(O_2) = 10^{-4}$ Torr. AFM topographic images of the film show that the one unit cell step-and-terrace structure of the substrate is preserved.

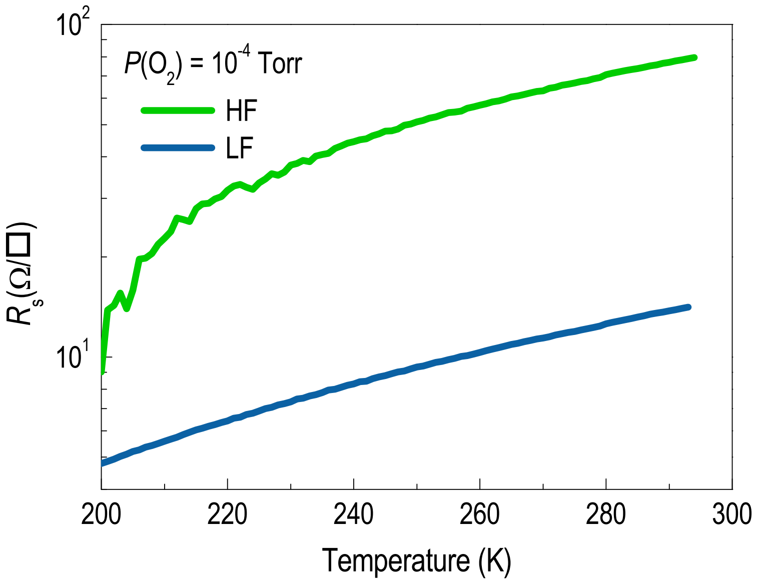

Supplementary Figure S2. The temperature-dependent sheet resistance, $R_s(T)$, of homoepitaxial $SrTiO_3$ films grown at $P(O_2) = 10^{-4}$ Torr with high and low oxygen flow rates (HF and LF). This result is consistent with the optical absorption spectra. The LF grown samples show more metallic nature compared to the HF grown sample due to larger oxygen vacancy concentration. We note that cation vacancies might also affect the charge transport behaviour in $SrTiO_3$ thin films, e.g., by trapping free electrons. However, in our study, the effect by the cation vacancies is much smaller than that of the oxygen vacancies which provides plenty of charge carriers.

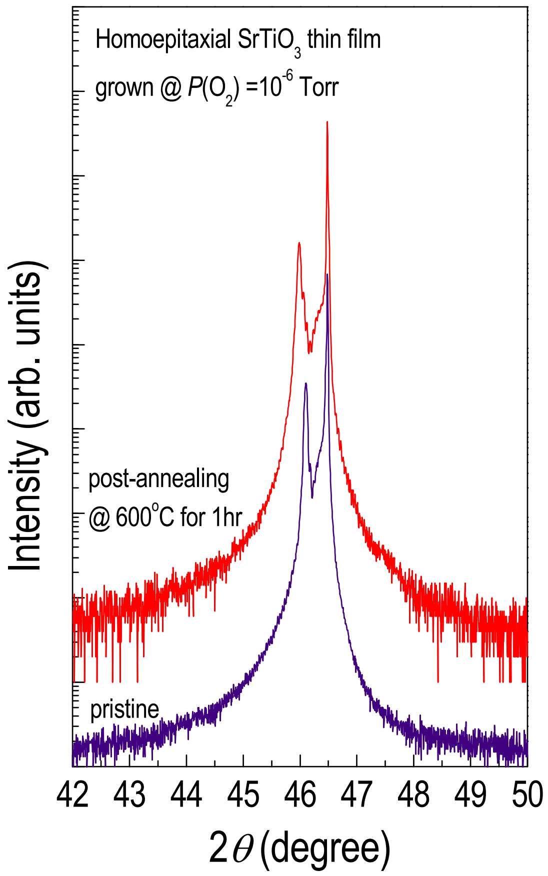

Supplementary Figure S3. X-ray diffraction *θ*-2*θ* scans of pristine and annealed $SrTiO_3$ thin films grown at $P(O_2) = 10^{-6}$ Torr. After post-annealing in air, the *c*-axis lattice constant of the $SrTiO_3$ thin film did not change significantly, indicating that oxygen vacancies does not induce lattice expansion in $SrTiO_3$.

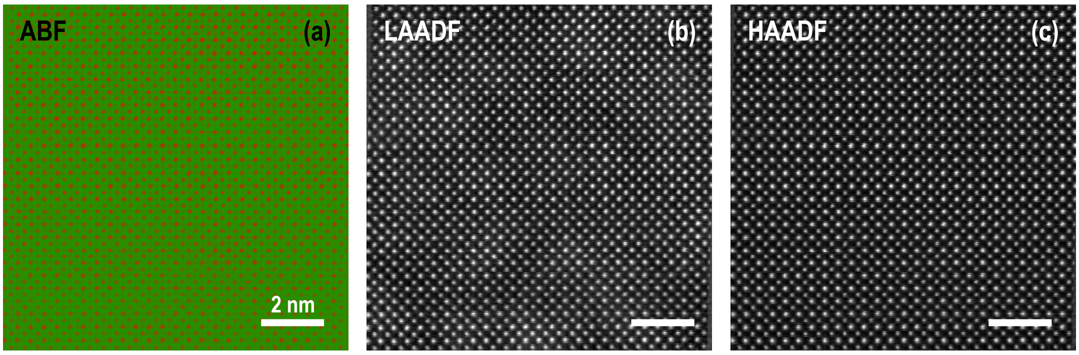

Supplementary Figure S4. STEM images of homoepitaxial $SrTiO_3$ thin film grown at $P(O_2) = 10^{-1}$ Torr. (a) ABF, (b) LAADF, and (c) HAADF-STEM images indicate single-crystalline thin film without any defects expected for stoichiometric $SrTiO_3$.

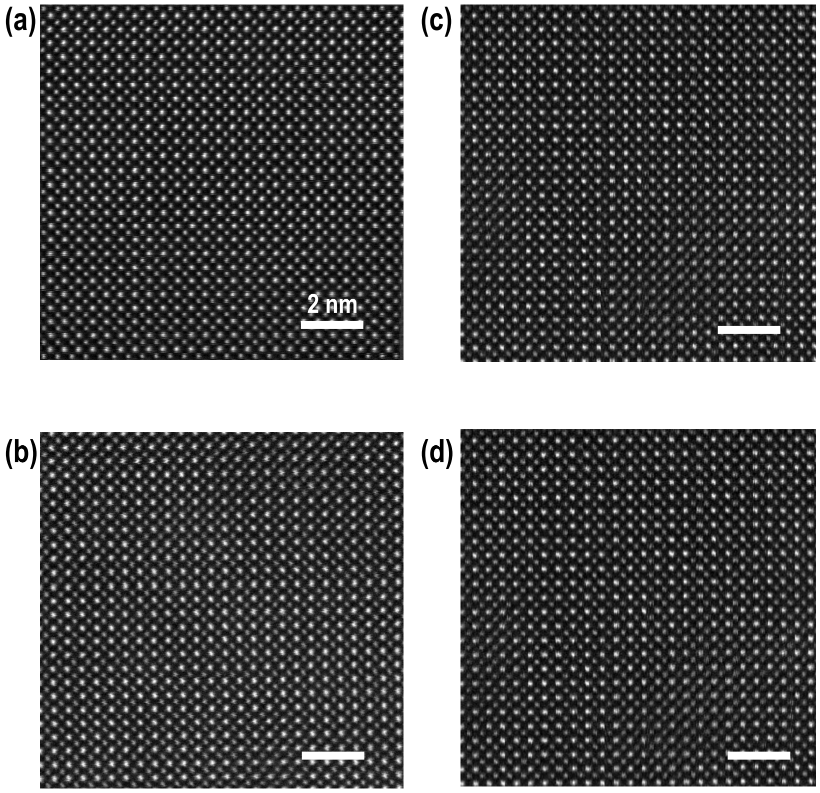

Supplementary Figure S5. HAADF-STEM images of homoepitaxial $SrTiO_3$ thin film. The HAADF images show no significant difference among the $SrTiO_3$ thin film grown at $P(O_2)$ = (a) 10^{-1} Torr, (b) 10^{-6} Torr, (c) 10^{-4} Torr with HF, and (d) 10^{-4} Torr with LF.

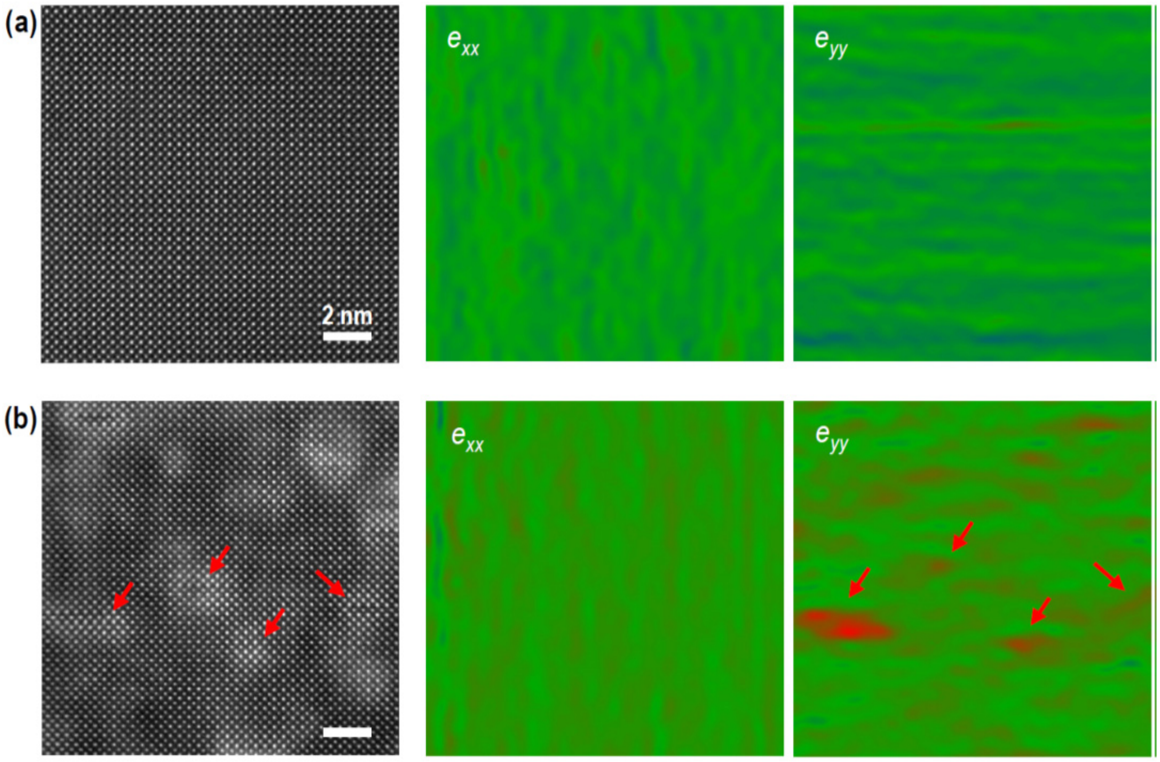

Supplementary Figure S6. Geometric phase analyses (GPA) of homoepitaxial $SrTiO_3$ thin films. LAADF-STEM and GPA images are shown for the corresponding spot for the films grown at $P(O_2)$ = (a) 10^{-1} and (b) 10^{-6} Torr. The film grown at low pressure shows substantial local lattice distortion due to vacancy cluster formation.
